# Training in Robotic Surgery—an Overview

**DOI:** 10.1007/s11934-017-0710-y

**Published:** 2017-06-24

**Authors:** Ashwin N. Sridhar, Tim P. Briggs, John D. Kelly, Senthil Nathan

**Affiliations:** 1 Department of Urology, University College London Hospital NHS Trust, London, UK; 2 Division of Surgery and Cancer, University College London, London, UK

**Keywords:** Robotic surgery, Urosurgery, Robotic surgical training

## Abstract

**Purpose of Review:**

There has been a rapid and widespread adoption of the robotic surgical system with a lag in the development of a comprehensive training and credentialing framework. A literature search on robotic surgical training techniques and benchmarks was conducted to provide an evidence-based road map for the development of a robotic surgical skills for the novice robotic surgeon.

**Recent Findings:**

A structured training curriculum is suggested incorporating evidence-based training techniques and benchmarks for progress. This usually involves sequential progression from observation, case assisting, acquisition of basic robotic skills in the dry and wet lab setting along with achievement of individual and team-based non-technical skills, modular console training under supervision, and finally independent practice.

**Summary:**

Robotic surgical training must be based on demonstration of proficiency and safety in executing basic robotic skills and procedural tasks prior to independent practice.

## Introduction

Robotic surgery has exponentially increased over the last decade. In 2015, there were more than 650,000 procedures performed all over the world [1]. In Europe, most robotic procedures were in urology, whereas in the USA, gynecology and general surgery led the field [1]. Specialties such as ENT [2] and thoracic surgery [3] are growth areas. The rapid adoption and dissemination of this technology have largely been as a result of the perceived benefits of improved ergonomics, dexterity, safety, and ease of surgery. Following the initial description by pioneers in this field, many established open and laparoscopic surgeons undertook robotic surgery without following a standardized, validated robotic curriculum [4]. The need for formal assessment of competency to ensure safe and sustained growth has led various groups to propose competency-based training programs in robotic surgery.

The robotic surgical platform represents a technological change, moving away from open surgery. Although viewed as an evolution of laparoscopic surgery, the skills required for novice robotic surgeons are for console control and maneuvers without haptic feedback, rather than those required for two-dimensional surgery using instruments within a restricted range of movement (as with laparoscopic surgery). A structured curriculum, which encompasses the acquisition of basic robotic skills as well as more complex maneuvers, allows the development of these skills in a safe and stepwise manner over a relatively short learning curve, [5 ^••^, 6 ^•^, 7].

Basic robotic surgical training can be divided into patient side training and console training. Patient side training incorporates patient positioning, establishing pneumoperitoneum, procedure specific port placement, robot docking and basic laparoscopic skills while console training encompasses dry and wet lab simulation and supervised operating. The development of non-technical skills to function in this multidisciplinary complex environment is an integral part of training and should occur in parallel with the development of technical skills.

This commentary sets out an evidence-based road map highlighting essential elements to be included within a curriculum for basic and advanced robotic training.

## Patient Side Training

Like any surgical procedure, development of robotic skills follows a progression of observing, assisting, performing under supervision, and finally independent practice. Patient side training has a two-pronged benefit. It not only exposes the trainee to the steps of the operative procedure (what to do when on the console), but also necessitates the development of skills unique to the assistant (troubleshooting at the patient side to make the procedure run smoothly). The assistant develops an understanding of the ergonomics and restriction of access created by the robotic arms. No metrics such as number of procedure or duration of patient side assistance are available. It is plausible that patient side skills are acquired relatively quickly, and that establishing a sign off of competency would enable progression to console in a relatively short duration.

### Patient Positioning and Port Placement

Patient positioning and port placement play a key role in the ergonomics of the procedure. Proper patient positioning not only ensures that each member of the surgical team (patient side assistant, scrub nurse, anesthetist) gets adequate access to the patient, but also maintains an optimal spatial configuration between the patient cart of the robot and the target organ in question. Similarly correct port placement enables access to target organs, allowing for the required triangulation, without any extracorporeal or intracorporeal instrument clashes. Simulation of this can be learnt in sham operating theaters placing ports into mannequins and testing for access and instrument clash and emergency undocking procedures.

### Achieving Basic Laparoscopic Skills

Basic laparoscopic skills required for a robotic surgeon include laparoscopic access and creation of pneumoperitoneum, take down of adhesions that prevent port insertion, application of clips, suction and retraction. These basic skills can be acquired in a dry lab and fine-tuned during bedside training.

The acquisition of basic laparoscopic skills has its own learning curve, but has been shown to ease the development of robotic skills on the console. Angell et al. [8] demonstrated this in a cohort of medical students in a dry lab setting. The task involved incising a spiral structure. The study showed that in a laparoscopic and robotic naive cohort, intensive training in basic laparoscopic skills reduced the time taken to perform the task robotically as well as reduced the number of errors. A similar study by Kilic et al. [9] showed that laparoscopic proficient gynecology residents performed robotic knot tying significantly better than laparoscopic naive residents.

A potential explanation for this benefit of basic laparoscopic skills is the development of a robust and safe method of instrument position and use. As laparoscopic instruments do not allow much freedom, there are limited ways of achieving a set task (say needle positioning). The procedural skills so developed tend to be the most ergonomic and safe way of performing the task. The increased maneuverability of the robotic platform in a sense allows development of many ways of achieving a task in a dry lab. All methods may not necessarily be the most ergonomic, and the skills developed in this setting may not be generalizable during a difficult operation. Hence, developing the correct basic laparoscopic skills provides a cognitive imprint that helps develop the correct robotic skills on the console, which can bail the surgeon out in difficult robotic cases.

Development of basic laparoscopic skills also improves spatial awareness (of organs as well as instruments), the ability to delicately handle tissues and the ability to safely maneuver and operate in three-dimensional space [10].

In addition to skill acquisition, development of these assistant skills (technical as well as non-technical) can serve as an early indicator of the future learning curve [11].

## Console Training

The current robotic system is a master slave system and the console is the interface controlling mechanical movement [12]. Similar to any advanced technological training, knowledge and working of the console is of paramount importance. Online modules are available that introduce the basic concepts of the currently available system (https://www.davincisurgerycommunity.com/Training?tab1=TR). Certification in these online modules is essential prior to embarking on any console training. Most modules such as describe each component of the system and also provide information on troubleshooting. Proficiency in these basic console skills (such as camera, pedal, finger control) can be achieved in a relatively straightforward manner in a dry lab or virtual reality (VR) simulated environment. Individual and team reaction to system errors can be simulated, repeated and assessed.

Advanced console skills (such as excision, suturing and use of diathermy) need to be developed in a mentored simulation environment, either undertaken in a VR simulator, dry lab or a wet lab (live animal/cadaveric animal or human models). With the evolution of competency-based training and a focus on patient safety in modern surgical practice, simulation provides not only a platform for initial skill development, but also skill assessment.

### Virtual Reality Simulators

#### Types of Simulators, Features and Validation

VR simulation is increasingly used in medical training and is considered a first and essential step in robotic surgical training. An advantage of VR systems software is the assessment and measurement of progress. Simulated exercises start from basic console control and increase in difficulty to complex tasks. There are five VR simulators currently available for robotic training. These include the Robotic Surgical Simulator (RoSS™; Simulated Surgical Systems, Buffalo, NY); dV-Trainer™ (Mimic Technologies, Inc., Seattle, WA); SEP Robot™ (SimSurgery™, Norway); the da Vinci Skills Simulator (Intuitive Surgical, Sunnyvale, CA) and more recently the Robotix mentor™ (3D systems, formerly Symbionix, Israel). All these simulators have been evaluated to have face validity (looks like what it simulates), content validity (accurately simulates the test condition) and construct validity (can differentiate between novice and expert except RoSS™) [13, 14, 15–22].

In addition to simulation of basic skills, most simulators now have procedure specific components, for example Hands-On Surgical Training or HOST™ in the RoSS™ [13] and Maestro AR in the dV-Trainer™ (http://www.mimicsimulation.com/products/maestro-ar/). These enable the trainee to register procedure specific movements required using tailored videos. The trainee sits at the console grasping the pincer grip and watches a video of the procedure (e.g., robotic prostatectomy) being performed, while the console arms move in accordance with the operating surgeon. Once the movements are registered, the trainee can mimic the movements of the surgeon and perform the procedure in real time. The movement of the trainee can be tracked and evaluated by the on board software. Chowriappa et al. [23] evaluated the role of the HOST™ skills training for robotic urethrovesical anastomosis in a randomized controlled trial. They found that participants who underwent the HOST™ skills training had better scores in the domains of needle driving (3.0 vs 2.3; *P* = 0.042), needle positioning (3.0 vs 2.4; *P* = 0.033) and suture placement (3.4 vs 2.6; *P* = 0.014). The HOST™ group obtained significantly higher scores (14.4 vs 11.9; *P* 0.012) on the GEARS score (Global evaluative assessment of robotic skills). They also found lower temporal demand and effort in the HoST group For 70% of participants, HoST the training experience was similar to a real surgical procedure and 75% of trainees responded that this training could improve confidence in performing a real procedure [23].

The latest generation dV-Trainer™ (http://www.mimicsimulation.com/products/xperience/) and Robotix mentor™ have a laparoscopic assistant component in parallel with the virtual reality console. These allow the console surgeon to develop advanced console procedural skills while the assistant develops their bedside skills, fostering greater teamwork. Team-based management of hazard scenarios and troubleshooting skills can be developed and assessed using these platforms.

#### VR Simulation to Improve Console Performance

Validation aside, it is important to see if use of the VR simulator actually improve performance on the console. Lerner et al. [24] showed that training with the dV-Trainer™ substantially improved performance in Pattern Cutting and Peg Board times (*P* = 0.04 and *P* = 0.006, respectively) on the dVSS (dry lab) when compared with training on the dVSS alone. The cohort consisted of two groups of residents, one that underwent training on the dV-Trainer™, and the other that trained on the dVSS using routine dry lab training. Hung et al. showed that training on VR simulators significantly improved performance on tissue exercises in trainees with low baseline robotic skills in a randomized controlled trial. However, this improvement was not significant in trainees with high baseline robotic skills, indicating the need for tailoring the curriculum to each trainee. Tergas et al. similarly demonstrated a significant improvement in “time to completion” and “economy of motion” for novices after training on the Da Vinci Skills Simulator.

Despite perceived advantages of robotic simulation training, little data exists on its predictive validity (transfer of skills into the Operating Room). Hung et al. [25] in a different study explored the concept of cross-method validation. They demonstrated that performance on box trainers correlated with performance on VR simulators, which in turn strongly correlated with in vivo tissue performance in wet labs. The next step of correlation between proficiency in the labs with performance in the operating rooms needs to be established. The next step of correlation between proficiency in the labs with performance in the operating rooms needs to be established.

Another important aspect of VR simulation-based training is its role in the maintenance/retention of acquired robotic skills. Lendvay et al. [26] showed that a warm up practice session on the VR simulator improved task performance and reduced errors in the dry lab not only for basic but also for complicated skills such as robotic suturing. The cohort included trainees and experts who had undergone the same robotic training, but were divided into two groups—one that did a warm up session, and the other that read a book in the meantime.

#### Setting a Benchmark for Attainment of Competency

Setting a benchmark for attainment of competency in VR skills is essential to progress to the next step of training. Raison et al. [27 ^•^] analyzed the participant and expert scores from European Association of Urology VR courses and identified five assessment exercises; three basic level exercises (Pick and Place, Camera Targeting 1, Peg Board 1) and two advanced level exercises (Suture Sponge and Thread the Rings 1). The three basic exercises tested fundamental robot skills including EndoWrist manipulation, clutching, three-dimensional vision, and camera control whereas the advanced exercises tested complex skill of suturing, requiring needle driving in addition to competent execution of basic robotic surgical skills. The technique of mean expert scores to gauge proficiency was used and potential benchmark standards for competency were set at 60, 75 and 90% of the mean expert score. Modeling against participant outcome data identified a competency standard of 75% of mean expert score as a suitable standard based on the performance of novice (no robotic surgical experience), intermediate (1–74 robotic procedures performed) and expert participants (>75 robotic procedures performed).

Noureldin et al. [28] defined competency based on the norm-referenced method, in which experts performed the tasks on the da Vinci Surgical Skills Simulator prior to the trainees, and a passing score was defined as the average of the experts’ total scores (MScore performance metrics inherent to the system) minus one standard deviation for each task. They incorporated VR simulation into the Canadian Objective Structured Clinical Examinations (OSCE) to assess the basic robotic skills of urology Post-Graduate Trainees (PGTs). Although their benchmark passed only one-third of their trainees, it was able to discriminate between novice and more experienced trainees.

Both of these studies demonstrate that benchmarks are not rigid scores that are set on the system, but rather need to be modifiable to the training institute where training is taking place.

### Dry Lab Training

Dry lab simulation is cost effective and can reliably simulate cutting, suturing and grasping exercises. As the user is actually sitting on the daVinci™ surgical system (dVSS) console and using robotic instruments to complete tasks, the fidelity of simulations is very high [29]. It simulates real time challenges and is a good interface to learn initial console troubleshooting, especially with regard to camera and clutch control, position of hands etc. The consumables for dry lab exercises can be as simple as routine beads/needles/sutures, to sophisticated vascular and bowel models. Dry lab exercises are however limited in that it is difficult to maintain a standardized record or method of assessment, something that is essential in the early stage. Objective assessments made by a keen observing trainer are required in order for the trainee to have any benefit from the system.

Siddiqui et al. [30 ^•^] developed and validated robotic objective structured assessments of technical skills (R-OSATS). Performance for each simulation drill (5 generic exercises) was assessed across four categories depth perception/accuracy, force/tissue handling, dexterity and efficiency. Each category was scored from 1 to 5, with higher scores indicating more proficiency. Scores are summed across categories, giving a maximum score of 20 per drill. Using this, they were able to demonstrate construct validity for R-OSATS. They were also able to set up benchmark scores for R-OSATS [31 ^••^] using the modified Angoff and the contrasting groups methods. Using these methods, the minimum score for competence was determined to be 14 per drill. To minimize the need for availability of expert assessors, the same team were able to demonstrate that crowd-sourced assessments of recorded dry lab surgical drills using R-OSATS were a suitable alternative [32].

### Wet Lab Training

Handling of tissues and understanding the reaction of tissues to instrument touch cannot be learnt in dry labs. Further, use of diathermy and vascular control can only be learnt in wet labs. Experience in the wet lab soon teaches the trainee to recognize the consistency of tissues based solely on visual clues. Robotic wet labs provide excellent training ground for near live surgical exposure. Wet labs can provide three different types of training material. Frozen animal parts [33], frozen human body parts [34] and live animals [35] with the cost increasing proportionately. More centers around the world are moving to animal body parts due to the cost factor. Animal and human body parts are excellent training material to learn handling of tissues, dissection, excision, diathermy and suturing techniques. Embalmed body parts allow vascular identification and dissection but alas do not provide a learning ground for vascular control.

Live anesthetized or euthanized animal models are expensive and limited with regard to number of times they can be used, but have an advantage of bleeding simulation. These limiting factors have resulted in low uptake in the early stages of a training program.

### Training in the Operating Room

Once signed off on simulated skills, the trainee should be competent to learn the steps of the procedure on the console safely. This should follow a modular approach. The role of a mentor is crucial in this process. The trainee must enter into an agreement with the mentor who will oversee training. The modular process begins with the trainee performing the simplest part of the procedure, and progressively taking on increasingly difficult bits as the mentor sees fit. The transition of mentor from preceptor (who will step in when required) to proctor (who supervises and allows the trainee sufficient opportunity to operate) usually indicates that a trainee is progressing.

Lovegrove et al. [6 ^•^] developed a safety and assessment tool to gauge the technical skills of surgeons performing robot-assisted radical prostatectomy during modular training based on Healthcare Failure Mode and Effect Analysis (HFMEA) methodology. The operative procedure was deconstructed to identify 17 steps and trainees undergoing modular training were assessed by their mentors from a score of 1–5. A score of 4 or above suggested attainment of competency. Using this they were able to monitor progress and define a learning curve for each step of the procedure.

Dual console dVSS, although coming at a substantial increase in cost, allows the mentor to step in and take over immediately without the trainee having to leave the console, potentially providing the trainee with more “operating time” on the console. Smith et al. [36] showed that this did not deviate significantly from single console procedures in terms of operative time and outcomes. Dual console training would be invaluable in vascular management.

Tele-mentoring [37] is an exciting and evolving technology that allows an expert to provide advice without physically being in the theater. The mentor can view the same images as the operating surgeon and provide expert guidance in real time. Though probably not realistic in its present state for early training, it could have a significant role in the future.

## Non-technical Skills Development for Robotic Surgery

The presence of the robot in the operating room poses a unique challenge to team communication and risk management and hence development of non-technical skills is of paramount importance. Studies in open surgery [38] have identified that 86% of adverse surgical events were due to “system errors” and were not related to technical skills. Overall, 40% of intraoperative errors were found to relate to failures in communication alone [38]. Non-technical skills such as teamwork, leadership, situational awareness, and decision-making have all been shown to have a significant impact on surgical success [39] and can be developed easily in a simulated environment. A recent systematic review by Wood et al. [40 ^••^] evaluated various non-technical skills training tools for both individual surgeon and team and concluded that NOTSS (Non-Technical Skills for Surgeons) was the gold standard training tool for the individual surgeon training whereas the Oxford NOTECHS (Nontechnical Skills Training Tools for the Surgical Team) II was the most favorable for team-based training.

## Discussion

The importance of having a validated training curriculum not only stems from the responsibility towards patient safety, but also from the ensuing issues with credentialing and associated liability. Proficiency-based training curricula that comprehensively address the skills necessary to perform robotic operations have shown construct and content validity as well as feasibility [41–44]. The initial years of the robotic era saw multiple versions of such a training curriculum individual to a center or group of centers. They ranged from 2 days to 10 weeks of training, were shown to be feasible and did show a measurable improvement in skills; however, they lacked uniformity in credentialing. Although validated for technical skills, these did not account for the development of non-technical/team skills essential for a novice surgeon.

More recently in 2012, a consortium of experts compiled a curriculum and outcome measures through a series of conferences to set up a Fundamentals of Robotic Surgery (FRS) program [45], akin to the Fundamentals of laparoscopic skills (FLS). The program consisted of a curriculum of didactic lectures, psychomotor skills labs, and team training activities. Twenty-five specific outcome measures are used to assess the achievement of robotic surgical skills. The proposed program aimed to establish a validated bar, after the attainment of which the trainee can build their skills with increasing hands-on experience in the operating room. Stegemann et al. [7] more recently showed in a randomized control trial that such a curriculum significantly improved basic robotic skills in those residents who undertook it, when compared to those who did not.

Ahmed et al. [5 ^••^] more recently designed a curriculum for robotic fellowship training based on structured focused group interviews among an international expert panel. Various iterations of content analysis of recorded interviews were discussed and analyzed and a curriculum was designed. A quantitative questionnaire about this curriculum was disseminated to attendees to assess the level of agreement with the designed curriculum. It takes the novice surgeon through online teaching, simulation in the laboratory environment, full immersive modular training at the workplace and final sign off of videoed full procedure and trainer’s report for certification. Although initially set at 3 months, the authors advocated at least 6–9 months of training in such a modular method. Individual sing off benchmarks were however not specified and were left to the discrimination of the trainer.

**Fig. 1:**
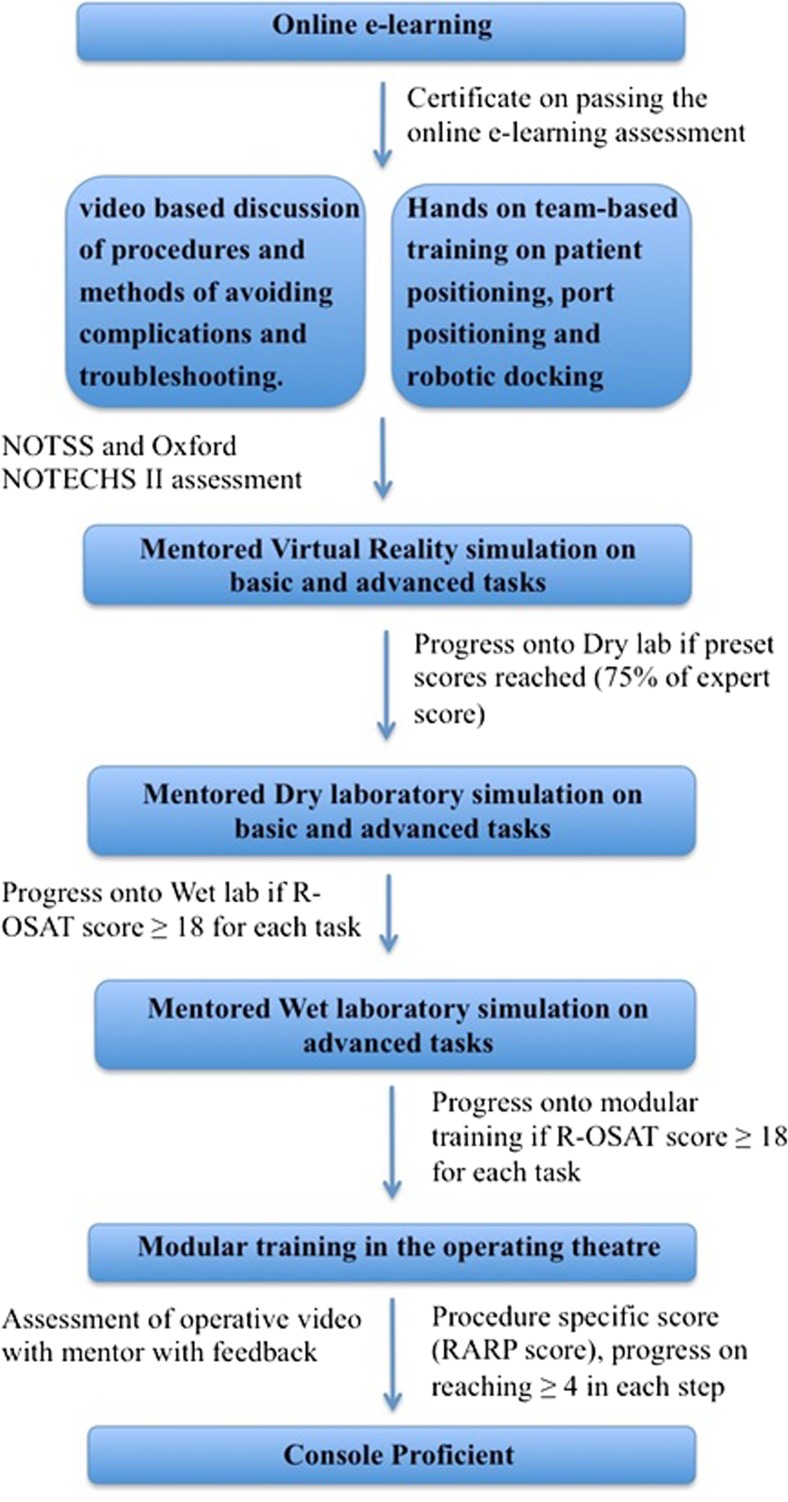
Pathway for training in robotic surgery

Collectively, a complete curriculum can be defined as in Fig. 1, based on the evidence generated in this review. Prospective evaluation of such a curriculum for robot-assisted radical prostatectomy for five novice surgeons at our center has demonstrated that patient outcomes are not adversely affected, and on the contrary, the learning curve for oncological and functional recovery (as defined as achievement of outcomes to the level of the trainers) can occur within the first 50 independent cases. The strength of implementation of such a curriculum lies in the background of a high volume of cases, qualified trainers, structured training schedules and regular assessment of skills.

## Conclusion

In conclusion based on our early experience, we would recommend a road map of training as delineated in Fig. 1 with assessment of competency at every level before progression. Such a training program would be based on demonstration of proficiency and safety in executing basic robotic skills and procedural tasks as well as achievement of non-technical skills in the practice laboratory prior to proceeding to modular training, and finally to sign off and independent practice.

## References

[1] Annual report 2015.

[2] Weinstein GS, OʼMalley BW, Desai SC, Quon H (2009). Transoral robotic surgery: does the ends justify the means?. Curr Opin Otolaryngol Head Neck Surg.

[3] A. Toker, Robotic thoracic surgery: from the perspectives of European chest surgeons. *J. Thorac. Dis.*, vol. 6 Suppl 2, no. Suppl 2, pp. S211-6, May 2014. 10.3978/j.issn.2072-1439.2014.05.05 PMC4032961 24868438

[4] van der Poel H, Brinkman W, van Cleynenbreugel B, Kallidonis P, Stolzenburg J-U, Liatsikos E, Ahmed K, Brunckhorst O, Khan MS, Do M, Ganzer R, Murphy DG, Van Rij S, Dundee PE, Dasgupta P (2016). Training in minimally invasive surgery in urology: European Association of Urology/International Consultation of Urological Diseases consultation. BJU Int.

[5] •• K. Ahmed, R. Khan, A. Mottrie, C. Lovegrove, R. Abaza, R. Ahlawat, T. Ahlering, G. Ahlgren, W. Artibani, E. Barret, X. Cathelineau, B. Challacombe, P. Coloby, M. S. Khan, J. Hubert, M. S. Michel, F. Montorsi, D. Murphy, J. Palou, V. Patel, P.-T. Piechaud, H. Van Poppel, P. Rischmann, R. Sanchez-Salas, S. Siemer, M. Stoeckle, J.-U. Stolzenburg, J.-E. Terrier, J. W. Thüroff, C. Vaessen, H. G. Van Der Poel, B. Van Cleynenbreugel, A. Volpe, C. Wagner, P. Wiklund, T. Wilson, M. Wirth, J. Witt, and P. Dasgupta, Development of a standardised training curriculum for robotic surgery: a consensus statement from an international multidisciplinary group of experts, *BJU Int.*, vol. 116, no. 1, pp. 93–101, Jul. 2015. **This study provides a structured pathway for training with validated development protocol. However, it fails to provide benchmarks for progression**. 10.1111/bju.12974 25359658

[6] • C. Lovegrove, G. Novara, A. Mottrie, K. A. Guru, M. Brown, B. Challacombe, R. Popert, J. Raza, H. Van der Poel, J. Peabody, P. Dasgupta, and K. Ahmed, Structured and modular training pathway for robot-assisted radical prostatectomy (RARP): validation of the RARP assessment score and learning curve assessment, *Eur. Urol.*, vol. 69, no. 3, pp. 526–535, Mar. 2016. **This study provides a structured modular pathway for training in RARP that can be modified for other robotic procedures**. 10.1016/j.eururo.2015.10.048 26585582

[7] Stegemann AP, Ahmed K, Syed JR, Rehman S, Ghani K, Autorino R, Sharif M, Rao A, Shi Y, Wilding GE, Hassett JM, Chowriappa A, Kesavadas T, Peabody JO, Menon M, Kaouk J, Guru KA (2013). Fundamental skills of robotic surgery: a multi-institutional randomized controlled trial for validation of a simulation-based curriculum. Urology.

[8] Angell J, Gomez MS, Baig MM, Abaza R. Contribution of laparoscopic training to robotic proficiency: J. Endourol; Jun. 2013. 10.1089/end.2013.0082 23527871

[9] Kilic GS, Walsh TM, Borahay M, Zeybek B, Wen M, Breitkopf D (2012). Effect of residents’ previous laparoscopic surgery experience on initial robotic suturing experience. ISRN Obstet Gynecol.

[10] Keehner MM, Tendick F, Meng MV, Anwar HP, Hegarty M, Stoller ML, Duh Q-Y (2004). Spatial ability, experience, and skill in laparoscopic surgery. Am J Surg.

[11] Louridas M, Quinn LE, Grantcharov TP (2016). Predictive value of background experiences and visual spatial ability testing on laparoscopic baseline performance among residents entering postgraduate surgical training. Surg Endosc.

[12] V. K. Narula and W. S. Melvin, Robotic Surgical Systems, in *Robotic Urologic Surgery*, London: Springer London, pp. 5–14.

[13] Kesavadas T, Stegemann A, Sathyaseelan G, Chowriappa A, Srimathveeravalli G, Seixas-Mikelus S, Chandrasekhar R, Wilding G, Guru K (2011). Validation of robotic surgery simulator (RoSS). Stud Health Technol Inform.

[14] Seixas-Mikelus SA, Kesavadas T, Srimathveeravalli G, Chandrasekhar R, Wilding GE, Guru KA (2010). Face validation of a novel robotic surgical simulator. Urology.

[15] Kenney PA, Wszolek MF, Gould JJ, Libertino JA, Moinzadeh A (2009). Face, content, and construct validity of dV-trainer, a novel virtual reality simulator for robotic surgery. Urology.

[16] Sethi AS, Peine WJ, Mohammadi Y, Sundaram CP (2009). Validation of a novel virtual reality robotic simulator. J Endourol.

[17] Seixas-Mikelus SA, Stegemann AP, Kesavadas T, Srimathveeravalli G, Sathyaseelan G, Chandrasekhar R, Wilding GE, Peabody JO, Guru KA (2011). Content validation of a novel robotic surgical simulator. BJU Int.

[18] Hung AJ, Patil MB, Zehnder P, Cai J, Ng CK, Aron M, Gill IS, Desai MM (2012). Concurrent and predictive validation of a novel robotic surgery simulator: a prospective, randomized study. J Urol.

[19] Whittaker G, Aydin A, Raison N, Kum F, Challacombe B, Khan MS, Dasgupta P, Ahmed K (2016). Validation of the RobotiX mentor robotic surgery simulator. J Endourol.

[20] T. Alzahrani, R. Haddad, A. Alkhayal, J. Delisle, L. Drudi, W. Gotlieb, S. Fraser, S. Bergman, F. Bladou, S. Andonian, and M. Anidjar, Validation of the da Vinci Surgical Skill Simulator across three surgical disciplines, *Can. Urol. Assoc. J.*, vol. 7, no. 7–8, p. 520, Jul. 2013. 10.5489/cuaj.419 PMC3713157 23914275

[21] Liss MA, Abdelshehid C, Quach S, Lusch A, Graversen J, Landman J, McDougall EM (2012). Validation, correlation, and comparison of the da Vinci Trainer ™ and the da Vinci Surgical Skills Simulator ™ using the Mimic ™ software for urologic robotic surgical education. J Endourol.

[22] Schreuder HWR, Persson JEU, Wolswijk RGH, Ihse I, Schijven MP, Verheijen RHM (2014). Validation of a novel virtual reality simulator for robotic surgery. Sci World J.

[23] Chowriappa A, Raza SJ, Fazili A, Field E, Malito C, Samarasekera D, Shi Y, Ahmed K, Wilding G, Kaouk J, Eun DD, Ghazi A, Peabody JO, Kesavadas T, Mohler JL, Guru KA (2015). Augmented-reality-based skills training for robot-assisted urethrovesical anastomosis: a multi-institutional randomised controlled trial. BJU Int.

[24] Lerner MA, Ayalew M, Peine WJ, Sundaram CP (2010). Does training on a virtual reality robotic simulator improve performance on the da Vinci surgical system?. J Endourol.

[25] Hung AJ, Jayaratna IS, Teruya K, Desai MM, Gill IS, Goh AC. Comparative assessment of three standardized robotic surgery training methods: BJU Int; Mar. 2013. 10.1111/bju.12045 23470136

[26] Lendvay TS, Brand TC, White L, Kowalewski T, Jonnadula S, Mercer LD, Khorsand D, Andros J, Hannaford B, Satava RM (2013). Virtual reality robotic surgery warm-up improves task performance in a dry laboratory environment: a prospective randomized controlled study. J Am Coll Surg.

[27] • N. Raison, K. Ahmed, N. Fossati, N. Buffi, A. Mottrie, P. Dasgupta, and H. Van Der Poel, Competency based training in robotic surgery: benchmark scores for virtual reality robotic simulation, *BJU Int.*, vol. 119, no. 5, pp. 804–811, May 2017. **This study provides important benchmarks for progression in VR simulation training**. 10.1111/bju.13710 27862825

[28] Noureldin YA, Stoica A, Kassouf W, Tanguay S, Bladou F, Andonian S (2016). Incorporation of the da Vinci Surgical Skills Simulator at urology Objective Structured Clinical Examinations (OSCEs): a pilot study. Can J Urol.

[29] Ramos P, Montez J, Tripp A, Ng CK, Gill IS, Hung AJ (2014). Face, content, construct and concurrent validity of dry laboratory exercises for robotic training using a global assessment tool. BJU Int.

[30] • N. Y. Siddiqui, M. L. Galloway, E. J. Geller, I. C. Green, H.-C. Hur, K. Langston, M. C. Pitter, M. E. Tarr, M. A. Martino, and C. to, Validity and reliability of the robotic objective structured assessment of technical skills HHS Public Access, *Obs. Gynecol*, vol. 123, no. 6, pp. 1193–1199, 2014. **This study provides important benchmarks for progression in drylab simulation training**. 10.1097/AOG.0000000000000288 PMC4352540 24807319

[31] •• N. Y. Siddiqui, M. E. Tarr, E. J. Geller, A. P. Advincula, M. L. Galloway, I. C. Green, H.-C. Hur, M. C. Pitter, E. E. Burke, and M. A. Martino, Establishing benchmarks for minimum competence with dry lab robotic surgery drills, 2016. **This study validates the benchmarks suggested previously**. 10.1016/j.jmig.2016.03.014 27013278

[32] M. R. Polin, N. Y. Siddiqui, B. A. Comstock, H. Hesham, C. Brown, T. S. Lendvay, and M. A. Martino, Crowdsourcing: a valid alternative to expert evaluation of robotic surgery skills, *Am. J. Obstet. Gynecol.*, vol. 215, no. 5, p. 644.e1–644.e7, Nov. 2016. 10.1016/j.ajog.2016.06.033 27365004

[33] Laguna MP, Arce-Alcazar A, Mochtar CA, Van Velthoven R, Peltier A, de la Rosette JJMCH (2006). Construct validity of the chicken model in the simulation of laparoscopic radical prostatectomy suture. J Endourol.

[34] Huri E, Ezer M, Chan E (2016). The novel laparoscopic training 3D model in urology with surgical anatomic remarks: fresh-frozen cadaveric tissue. Türk Üroloji Dergisi/Turkish J Urol.

[35] Wagner A, Munter M, Makarov D, Nielsen M, Scorpio D, Kavoussi LR (2008). Totally laparoscopic creation of a novel stapled orthotopic neobladder in the porcine model. J Endourol.

[36] Smith AL, Scott EM, Krivak TC, Olawaiye AB, Chu T, Richard SD (2013). Dual-console robotic surgery: a new teaching paradigm. J Robot Surg.

[37] Santomauro M, Reina GA, Stroup SP, L’Esperance JO (2013). Telementoring in robotic surgery. Curr Opin Urol.

[38] Gawande AA, Zinner MJ, Studdert DM, Brennan TA (2003). Analysis of errors reported by surgeons at three teaching hospitals. Surgery.

[39] Yule S, Flin R, Paterson-Brown S, Maran N (2006). Non-technical skills for surgeons in the operating room: a review of the literature. Surgery.

[40] •• T. C. Wood, N. Raison, S. Haldar, O. Brunckhorst, C. McIlhenny, P. Dasgupta, and K. Ahmed, Training tools for nontechnical skills for surgeons—a systematic review, *J. Surg. Educ.*, Dec. 2016. **This study is an excellent review of the non-technical skills assessment required for training**. 10.1016/j.jsurg.2016.11.017 28011262

[41] Suh I, Mukherjee M, Oleynikov D, Siu K-C. Training program for fundamental surgical skill in robotic laparoscopic surgery: Int. J. Med. Robot; Jun. 2011. 10.1002/rcs.402 21688381

[42] Tausch TJ, Kowalewski TM, White LW, McDonough PS, Brand TC, Lendvay TS (2012). Content and construct validation of a robotic surgery curriculum using an electromagnetic instrument tracker. J Urol.

[43] Arain NA, Dulan G, Hogg DC, Rege RV, Powers CE, Tesfay ST, Hynan LS, Scott DJ (2012). Comprehensive proficiency-based inanimate training for robotic surgery: reliability, feasibility, and educational benefit. Surg Endosc.

[44] Dulan G, Rege RV, Hogg DC, Gilberg-Fisher KK, Tesfay ST, Scott DJ (2012). Content and face validity of a comprehensive robotic skills training program for general surgery, urology, and gynecology. Am J Surg.

[45] R. Smith, V. Patel, S. Chauhan, and R. Satava, Fundamentals of robotic surgery: outcomes measures and curriculum development, ncsaglobal.com, 2012. 10.1002/rcs.1559 24277315

